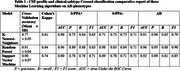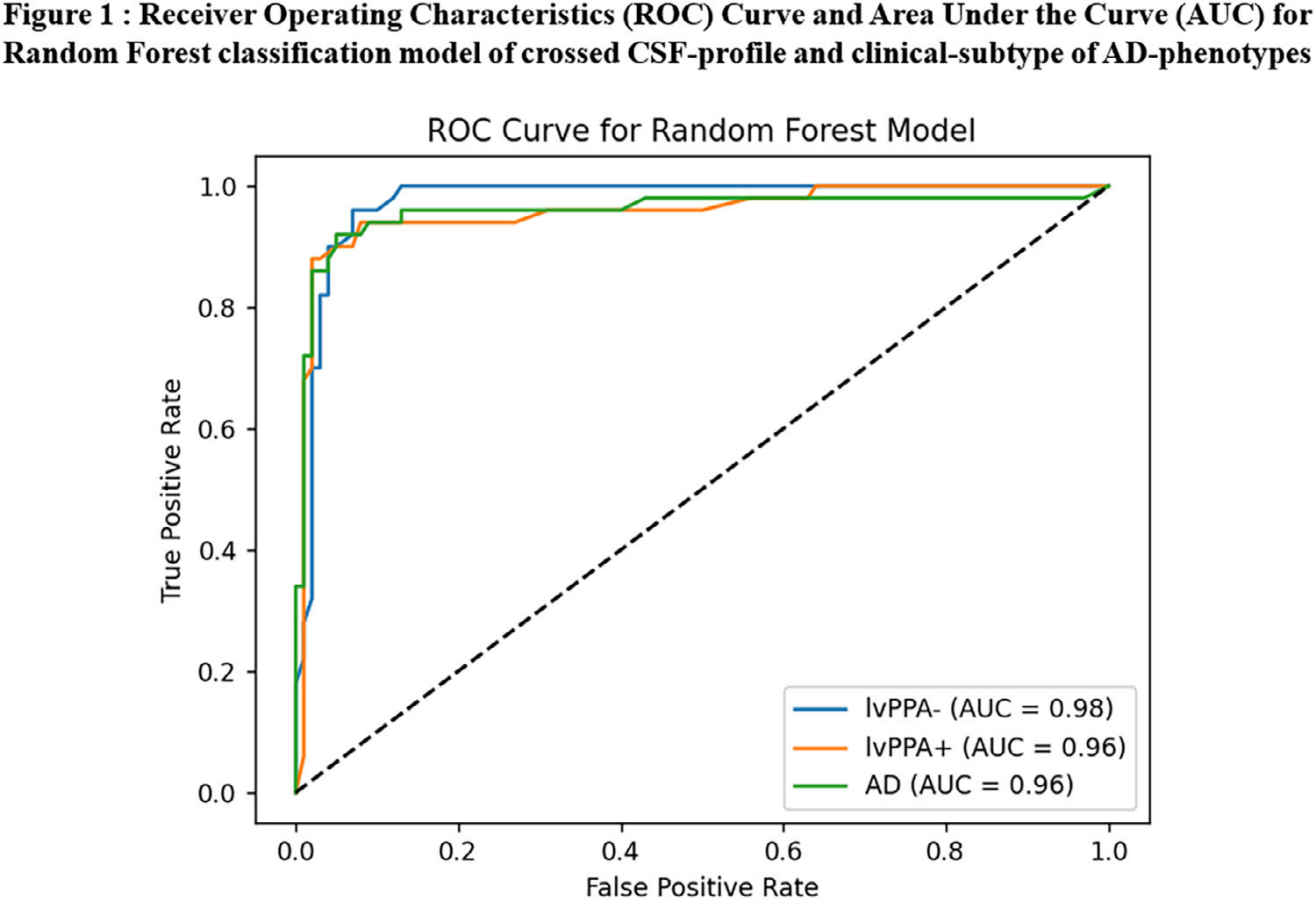# Speech‐based machine learning for early Alzheimer's phenotypes classification: Predicting both clinical diagnosis and underlying pathology at onset time

**DOI:** 10.1002/alz70863_110666

**Published:** 2025-12-23

**Authors:** Eloïse DA CUNHA, Valeria Manera, Raphael Zory, Auriane Gros

**Affiliations:** ^1^ Speech and Language Pathology Department, Université Côte d'Azur, Nice, Alpes Maritimes France; ^2^ CoBTeK Laboratory (Cognition Behaviour Technology), Nice, Alpes Maritimes France; ^3^ Interdisciplinary Institute of Artificial Intelligence, Université Côte d'Azur, Nice, Alpes Maritimes France; ^4^ University Hospital Center of Nice, Nice, Alpes Maritimes France; ^5^ Université Côte d'Azur, LAMHESS laboratory (Laboratoire Motricité Humaine Expertise Sport Santé), Nice, Alpes Maritimes France

## Abstract

**Background:**

Alzheimer's disease (AD) manifests through diverse clinical forms, each necessitating specific care approaches. Detecting both underlying pathology and precise clinical phenotype is critical to address the full spectrum of symptoms and provide tailored care. Logopenic variant of primary progressive aphasia (lvPPA) evolutive prognosis is particularly challenging. This syndrome presents dominant language AD‐like symptoms with different underlying pathologies such as AD or frontotemporal‐lobar‐degeneration (FTLD). Current diagnosis requires a multidisciplinary approach, combining CSF (Cerebro‐Spinal Fluid) analysis, imaging, and psychometric assessments. Speech markers were identified as valuable tools for distinguishing clinical diagnoses. This study evaluates their potential for cross‐classifying AD‐phenotypes with CSF‐profiles, providing a non‐invasive method for early prognosis of neurodegenerative evolution.

**Method:**

This study included 42 patients classified into three groups based on clinical phenotype and CSF biomarker profiles: lvPPA‐ (*n* = 10, underlying FTLD), lvPPA+ (*n* = 12, underlying AD), and AD (*n* = 20). Participants completed a 14 sentence repetitions task at diagnostic stage. Speech recordings were analyzed at both patient and sentence‐specific levels. Prosodic and temporal features were extracted and used to train supervised machine learning models (Random Forest, K‐Nearest Neighbors, and Support Vector Machine). Then, these models were parameterized to classify both clinical phenotypes and underlying pathologies of AD phenotypes. Cross‐validation (70/30 split) and hyperparameter tuning optimized model performance.

**Results:**

The three groups exhibited similar demographic profiles but significantly distinct clinical and pathological characteristics. Among the models tested, Random Forest demonstrated the most efficient and balanced performance, achieving a cross‐validation accuracy of 91%. It differentiated lvPPA‐, lvPPA+, and AD groups, with precision scores outperforming other models. These findings suggest that speech‐based classification can serve as a valuable tool for an early prognostic identification of both clinical phenotypes and underlying pathology, offering a non‐invasive tool for refining diagnostic precision at early‐stage of Alzheimer's disease trajectories.

**Conclusions:**

Speech‐based classification provides valuable early prognostic insights by linking clinical phenotypes with CSF profiles at onset time. By enhancing prognostic precision, such tools could improve early intervention strategies to counter disease progression. However, larger studies and advanced classification approaches are essential to confirm these findings and validate speech classification as a reliable, non‐invasive tool for early differential prognosis.